# Is R_2_* a New MRI Biomarker for the Progression of Parkinson’s Disease? A Longitudinal Follow-Up

**DOI:** 10.1371/journal.pone.0057904

**Published:** 2013-03-01

**Authors:** Miguel Ulla, Jean Marie Bonny, Lemlih Ouchchane, Isabelle Rieu, Beatrice Claise, Franck Durif

**Affiliations:** 1 CHU Clermont-Ferrand, Service de Neurologie A, Clermont-Ferrand, France; 2 University Clermont 1, EA3845, Clermont-Ferrand, France; 3 INRA, UR370 Qualité des Produits Animaux, Saint Genès Champanelle, France; 4 CHU Clermont-Ferrand, Pôle Santé Publique Médecine Légale Qualité Vigilances, Unité de Biostatistique Informatique Médicale et Technologies de Communication, Clermont-Ferrand, France; 5 University Clermont1, Laboratoire de Biostatistique Informatique Médicale et Technologies de Communication, Clermont-Ferrand, France; 6 CHU Clermont-Ferrand, Service de Radiologie B, Clermont-Ferrand, France; Philadelphia VA Medical Center, United States of America

## Abstract

**Purpose:**

To study changes of iron content in basal ganglia in Parkinson’s disease (PD) through a three-year longitudinal follow-up of the effective transverse relaxation rate R_2_*, a validated MRI marker of brain iron content which can be rapidly measured under clinical conditions.

**Methods:**

Twenty-seven PD patients and 26 controls were investigated by a first MRI (t_0_). Longitudinal analysis was conducted among the 18 controls and 14 PD patients who underwent a second MRI (t_1_) 3 years after. The imaging protocol consisted in 6 gradient echo images obtained at different echo-times for mapping R_2_*. Quantitative exploration of basal ganglia was performed by measuring the variation of R_2_* [R_2_*(t_1_) – R_2_*(t_0_)] in several regions of interest.

**Results:**

During the three-year evolution of PD, R_2_* increased in Substantia nigra (SN) (by 10.2% in pars compacta, p = 0.001, and 8.1% in pars reticulata, p = 0.013) and in the caudal putamen (11.4%, p = 0.011), without significant change in controls. Furthermore, we showed a positive correlation between the variation of R_2_* and the worsening of motor symptoms of PD (p = 0.028).

**Conclusion:**

Significant variation of R_2_* was longitudinally observed in the SN and caudal putamen of patients with PD evolving over a three-year period, emphasizing its interest as a biomarker of disease progression. Our results suggest that R_2_* MRI follow-up could be an interesting tool for individual assessment of neurodegeneration due to PD, and also be useful for testing the efficiency of disease-modifying treatments.

## Introduction

Increased iron concentration was found in specific brain structures of patients suffering from Parkinson’s disease (PD). Indeed, *post mortem* histological analysis [1], *in vivo* magnetic resonance imaging (MRI) [2]–[7] and transcranial sonography [8] studies are in agreement, highlighting iron deposition in the substantia nigra (SN) of PD patients, although results are controversial for other basal ganglia (BG) structures such as the putamen [9]–[11]. Whereas no association was shown between iron deposition and disease duration, clinical scores correlated with SN iron load in PD patients [4], [5], [12], suggesting that the amount of SN iron could be a biomarker of disease severity.

It is noteworthy that since there is no longitudinal data available in PD, all these results come from cross-sectional studies that do not directly assess changes in the amounts of iron in the brains of subjects over time. Thus, to further investigate the link between brain iron changes and PD progression, a longitudinal approach involving both PD patients and normal subjects appears to be the most appropriate study design for distinguishing between the concomitant effects of normal aging and disease evolution over time. Indeed, the amount of iron increases particularly in the caudate nucleus, putamen [13]–[16] and cerebral cortex [17] during normal aging.

Magnetic resonance imaging (MRI) is a non invasive imaging modality which makes it interesting for conducting longitudinal follow-ups. Moreover, it is a powerful tool for detecting iron deposits in the brain. Iron deposition induces local, fluctuating and non-fluctuating, magnetic field inhomogeneities which lead to faster signal decay and thus to an increase of the relating relaxation rates [18]. First, the relaxation rate R_2_ (R_2_ = 1/T_2_) is influenced by the effects of fluctuating microscopic magnetic field due to iron. The measurement of R_2_ is based on spin-echoes which discard the non-fluctuating magnetic field inhomogeneities whereas the fluctuating ones are preserved. It explains why iron effects are detectable through R_2_ variations. Secondly, the relaxation rate R_2_’ conveys the other non-fluctuating magnetic field effects due to iron. The latter are also measurable through R_2_’ variations using gradient-echoes [19]. It has been shown that R_2_ and R_2_’ exhibit a strong correlation with iron concentration, with similar sensitivities [20]. Since reversible and irreversible iron effects cumulate through R_2_*, which sums these two relaxation rates, this parameter is characterized by a higher sensitivity to iron content. This is why we propose to assess R_2_* as an imaging biomarker of PD evolution.

To achieve this, a 3-year follow-up longitudinal study was conducted on cohorts of PD patients and controls for dissociating the effects of disease progression and normal aging in regional variations of R_2_* in basal ganglia (BG).

## Methods

### Subjects

Twenty-seven PD patients were included in the study. They were recruited consecutively, among patients who presented themselves for consultation in the Movement Disorder unit of Clermont-Ferrand, France. All the patients were suffering from idiopathic Parkinson’s disease according to the criteria of the “Parkinson’s Disease Society Brain Bank” [21]. In addition, twenty-six control subjects were included, recruited among relatives of patients, who were all free of any history of neurological or psychiatric diseases.

All the subjects were interviewed regarding their previous medical history and a Mini Mental Status examination was performed to exclude demented subjects (MMS<26/30). In the PD group, disease duration, levodopa equivalent dose (LED) [22], Hoehn and Yahr (HY) stages and the motor part of Unified Parkinson’s Disease Rating Scale (UPDRS III) [23], were noted, the latter two being assessed in the “on” state. The lateralized UPDRS motor score on each side was also calculated (sum of items 20 to 26).

All the subjects were investigated by an initial MRI session (called t0). Three years later the subjects were contacted by mail or phone to participate in a second MRI session, to enable us to conduct a longitudinal analysis. After acceptance, 18 controls and 14 PD patients underwent this second MRI session (called t1). Thus 13 PD patients (1 death due to heart attack, 3 benefiting from deep-brain stimulation, 1 refusal and 8 moves) and 8 controls (unavailability) did not have the second MRI.

All subjects gave written consent and the study was approved by the local research ethics committee (Comité de Protection des Personnes Sud-Est, file AU 867).

### MRI Investigations

Images were obtained using a 1.5 T MRI system (Sonata, Siemens, Germany). The subjects were immobilized in the head coil for both emission and reception, by foam pads to reduce involuntary head movements. Disposable ear protectors were also used to reduce acoustic noise.

A T_1_-weighted sagittal scout image was first acquired to locate the anterior and posterior commissure (AC and PC). Then, an anatomical protocol was used to highlight BG structures in 14 slices acquired parallel to the AC-PC line.

Finally, we used a 2D steady-state free precession gradient echo sequence for mapping the apparent proton transverse relaxation rate (R_2_* = 1/T_2_*). For each of the 14 slices of the anatomical dataset, images were obtained at 6 different echo times (TE) ranging from 7.61 ms to 50 ms (7.61, 10, 20, 30, 40, 50 ms). The other image parameters were: TR = 1050 ms, FOV = 280×280 mm^2^, matrix = 128×128, slice thickness = 2.5 mm (volume of the resulting voxel 2.2×2.2×2.5 mm^3^). The duration of the whole MRI session was 40 minutes.

### Image Analysis

**Figure 1:**
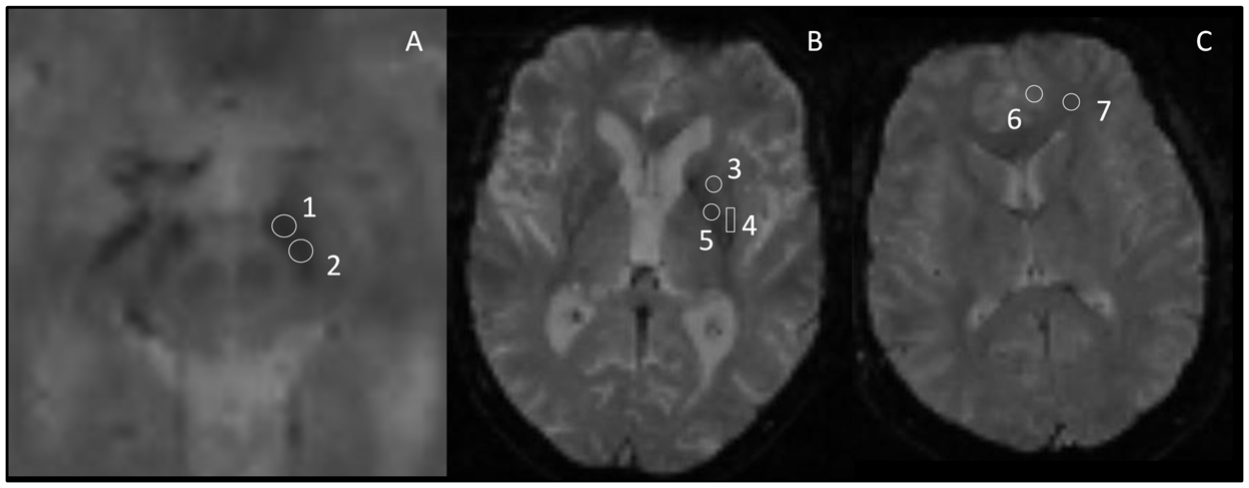
ROI location in three different slices (A, B and C) in anatomic images. TR = 1100 ms, TE = 50 ms, field-of-view (FOV) = 280×280 mm, acquisition matrix = 256×256 1: Substantia nigra pars reticulata (SNr). 2: Substantia nigra pars compacta (SNc). 3: rostral putamen (rPut). 4: caudal putamen (cPut). 5: Globus Pallidus (GP). 6: gray matter (GM). 7: white matter (WM).

Regions of interest (ROI) in which R_2_* was measured were defined manually on the anatomical images using ImageJ, a public domain software application (http://rsb.info.nih.gov/ij/). This approach was preferred to automatic segmentation because it is the only method able to subdivide SN into two parts (pars reticulata and pars compacta) [24]. The investigator responsible for ROI definition (M.U.) was unaware of subject group identification. The accurate position of each ROI was systematically controlled in the images obtained at the different TE and for the two MRI of the same subject based on relative distance of the ROI to the boundaries of the anatomical structure.The shape and size of the ROI were identical for a given structure in all subjects. Three BG structures were selected (Figure 1): the SN divided into two parts: pars reticulata and pars compacta (SNr and SNc respectively); according to recent data [5]; the putamen, also divided into two parts: rostral and caudal (rPut and cPut respectively); and the globus pallidus (GP). Grey matter (GM) and white matter (WM), both in the frontal lobe, were also studied. All ROI were placed bilaterally.

R_2_* values from the different brain structures were obtained by fitting the ROI-averaged magnitude of the six echoes with a single exponential decay using a nonlinear least-squares numerical procedure.

### Statistical Analysis

PD patients were compared to controls regarding gender (using Fisher’s exact test) and continuous characteristics such as age and period between t_0_ and t_1_ examinations (using the Wilcoxon test).

R_2_* values measured in the left and right hemispheres (in controls) as well as in the more and less affected side (in PD patients) in each brain structure were not significantly different (paired signed rank tests). Therefore these two measurements were averaged to obtain a single representative value in each structure. Quantitative variables were transformed into ordinal classes (age in quartiles, disease duration, HY stage and UPDRS in terciles) for inclusion as factors in ANOVAs. The effect of different variables on R_2_* at t_0_ was tested separately for each ROI and each group by analysis of variance (ANOVA); i.e. gender and age for all groups of subjects, and disease duration, HY stage and UPDRS for the PD group only. In the case of significant effect, a post-hoc multiple comparisons procedure was performed (Tukey test).

Subjects who benefited from two MRI sessions (n = 32) were used to assess the possible difference between R_2_* values by performing a signed rank test after computation of ΔR_2_* defined as the difference R_2_*(t_1_) – R_2_*(t_0_).

Furthermore, disease effect was tested for both R_2_* at t_0_ and ΔR_2_* through ANOVA involving PD patients (PD) and controls.

Finally, correlations between ΔR_2_* and variations of HY stages, ΔHY (HY*(t_1_) – HY*(t_0_)), variations of LED, ΔLED (LED (t_1_) – LED (t_0_)) and percentage changes of UPDRS III worsening (ΔUPDRS, %) were tested using a Spearman correlation.

All these statistical tests were performed on SAS (SAS v9.1, SAS institute inc., Cary, NC, USA) with a 0.05 type I error.

## Results

### Subjects

Table 1 summarizes the demographic and clinical characteristics of subjects.

**Table 1:** Demographic and clinical characteristics of subjects.

group	n (t_0_)	age(years)	gender(M/F)	disease duration (years)	HY stage (t_0_)	UPDRS III(t_0_)	n (t_1_)	delay between2 MRI (months)	HYstage (t_1_)	UPDRS III(t_1_)
**PD** **patients**	27	60.2±10.7	13/14	5.7±4.4	1.9±0.7	12.1±8.5	14	36.9±4.7	2.5±0.5*	19.8±14.7**
**controls**	26	57.0±8.5	9/17	NA	NA	NA	18	37.2±3.7	NA	NA

Mean ± standard error. M = male; F = female; NA = not applicable; HY = Hoehn and Yahr; UPDRS III = motor part of UPDRS (Unified Parkinson’s Disease Rating Scale).

* p<0.01 and **p<0.0001, vs. t_0_ (signed rank test).

When comparing the characteristics of control and PD groups, no significant difference was found in age (p = 0.314), gender (p = 0.264) and in the period between the two MRI sessions (p = 0.819).

The LED was 614±317 mg at t_0_ and 907±443 mg at t_1_, with a significant increase between t_0_ and t_1_ (p = 0.019). The HY scale and the UPDRS III in PD patients followed-up longitudinally were significantly higher at t_1_ compared to t_0_, reflecting disease evolution (HY, p = 0.007; UPDRS, p<0.0001).

The comparisons between subjects investigated only at t_0_ and subjects investigated twice did not reveal any significant difference regarding age (p = 0.667 for controls and p = 1 for PD patients) and gender (p = 0.118 for controls and p = 1 for PD patients) in all subjects, either regarding disease duration (p = 0.099), HY stage (p = 0.506) and UPDRS III (p = 0.101) in PD patients.

It should be noted that anatomical MRI sequences in all our subjects did not display any anomalies, in particular vascular lesions.

### Cross-sectional Analysis of R_2_*

Measurements performed at t_0_ on controls showed that R_2_* was higher in older subjects (Table 2). The effect of age turned out to be significant in the cPut (f = 5.39, p = 0.007) and the GM (f = 4.39, p = 0.016). Post-hoc analysis showed that the difference was significant between first three quartiles and last quartile, in both cPut and GM. Moreover, no effect of gender on R_2_* was found.

**Table 2:** R_2_ * (s^−1^) at t_0_ in each ROI (mean ± standard error).

		t/q	n	GM	GP	SNc	SNr	WM	rPut	cPut
**controls**	**age**	1	8	12.46±0.34	25.81±0.59	21.47±0.53	25.79±0.62	17.13±0.32	18.98±0.57	21.67±0.35
**(n = 26)**		2	7	11.61±0.53	23.73±0.67	20.56±0.52	25.23±0.91	16.25±0.32	18.74±0.70	20.96±0.66
		3	6	12.10±0.28	24.45±1.15	19.66±0.48	23.87±0.81	16.38±0.76	19.41±1.13	21.22±0.80
		4	5	14.03±0.68	24.97±1.32	20.89±0.86	25.45±1.35	17.18±0.52	20.33±0.86	26.17±1.55
	*F*			*4.39*	*0.6*	*1.29*	*1.02*	*1.25*	*0.69*	*5.39*
	*p*			*0.0165* *	*0.6237*	*0.3072*	*0.4063*	*0.3208*	*0.5708*	*0.0074* *
	**gender**	F	18	12.56±0.36	25.03±0.54	20.91±0.39	25.07±0.43	16.60±0.27	19.67±0.42	22.82±0.71
		M	8	12.19±0.35	24.21±0.82	20.20±0.41	25.28±1.10	17.01±0.49	18.38±0.80	20.92±0.49
	*F*			*0.15*	*0.51*	*0.12*	*0.88*	*2.46*	*2.48*	*0.51*
	*p*			*0.702*	*0.4858*	*0.7286*	*0.3612*	*0.1331*	*0.1316*	*0.4827*
**PD** **patients**	**age**	1	8	11.65±0.54	25.93±0.72	23.60±0.69	27.14±0.82	15.81±0.49	18.44±0.46	20.34±0.90
**(n = 27)**		2	5	12.12±0.70	25.87±1.61	22.27±0.61	27.43±2.24	17.25±0.61	18.20±0.51	22.63±1.50
		3	7	12.34±0.62	24.07±0.53	21.11±0.64	25.44±0.89	16.34±0.46	18.40±0.70	22.09±1.07
		4	7	11.54±0.87	25.07±1.19	23.32±0.76	28.26±1.08	15.29±0.55	22.33±1.17	24.91±1.46
	*F*			*0.22*	*0.07*	*1.26*	*1.32*	*3.08*	*4.4*	*1.76*
	*p*			*0.8787*	*0.9753*	*0.3225*	*0.3028*	*0.0575*	*0.0194* *	*0.1944*
	**gender**	F	14	12.15±0.48	24.18±0.44	22.54±0.61	25.54±0.50	16.00±0.36	19.37±0.74	22.23±0.97
		M	13	11.61±0.47	26.33±0.81	22.74±0.48	28.66±0.94	16.17±0.44	19.42±0.71	22.59±0.93
	*F*			*0,01*	*1,52*	*0,1*	*2,12*	*0,6*	*0,57*	*1,54*
	*p*			*0,9081*	*0,2361*	*0,7576*	*0,1649*	*0,4506*	*0,4625*	*0,2327*
	**diseases duration**	1	10	11.83±0.53	25.54±0.74	22.64±0.71	26.71±0.51	15.36±0.38	18.65±0.80	22.09±1.32
		2	9	11.40±0.46	25.55±1.02	23.12±0.54	26.48±0.79	16.61±0.37	20.57±0.92	22.86±1.13
		3	8	12.51±0.74	24.44±0.80	22.07±0.77	28.09±1.74	16.39±0.65	18.99±0.82	22.29±0.99
	*F*			*0.17*	*0.74*	*0.02*	*2.02*	*3.16*	*1.02*	*1.37*
	*p*			*0.8464*	*0.4936*	*0.983*	*0.1647*	*0.0696*	*0.3831*	*0.282*
	**Hoehn and Yahr**	1	9	11.81±0.58	24.31±0.47	22.23±0.79	26.68±0.50	15.83±0.43	18.78±0.97	21.85±1.39
		2	9	11.54±0.43	26.33±0.70	23.12±0.48	27.78±0.93	16.06±0.41	19.73±0.64	22.32±0.85
		3	9	13.34±0.89	22.85±0.73	21.62±0.98	24.99±1.55	16.66±0.76	19.34±1.62	23.83±1.71
	*F*			*1.98*	*2.37*	*0.15*	*0.43*	*1.09*	*0.07*	*1.01*
	*p*			*0.1708*	*0.1257*	*0.8614*	*0.6598*	*0.3594*	*0.9285*	*0.3875*
	**UPDRS III**	1	8	12.40±0.54	25.65±0.88	23.05±0.82	27.07±1.51	16.49±0.97	19.96±0.87	22.89±1.16
		2	10	11.99±0.51	25.63±0.74	22.64±0.65	27.05±0.57	15.79±0.47	18.04±0.29	20.95±1.05
		3	9	11.31±0.67	24.37±0.95	22.25±0.59	27.00±1.13	16.03±0.58	20.39±1.15	23.58±1.16
	*F*			*1.21*	*0.35*	*0.03*	*0.18*	*0.71*	*1*	*0.96*
	*p*			*0.3227*	*0.7077*	*0.9674*	*0.8399*	*0.5077*	*0.3883*	*0.4034*

n = number of subjects. Age was divided into quartiles (q) (the classes for controls being 42–49, 50–53, 54–67, 68–76; for PD patients 42–49, 50–59, 60–68, 69–78) and disease duration, Hoehn and Yahr stage and UPDRS III in terciles (t) (the classes for disease duration being 1–3, 4–6, 7–21; for HY 1–1.5, 2, 2.5–4; for UPDRS III 4–7.5, 8–12.5, 13–40).

GM = gray matter; GP = Globus Pallidus; SNc = Substantia nigra pars compacta; SNr = Substantia nigra pars reticulata; WM = white matter; rPut = rostral putamen; cPut = caudal putamen.

* p<0.05; analysis of variance (ANOVA). Post-hoc multiple comparisons (Tukey test) showed in the three cases that the difference was significant between the three first quartiles and the fourth.

In PD patients, a significant effect of age in the rPut (f = 4.4, p = 0.019) was observed. Post-hoc comparisons revealed higher R_2_* values in the fourth quartile of age compared to the others. We also showed a trend of lower R_2_* in WM (f = 3.08, p = 0.057). Gender, disease duration, HY stage and UPDRS III did not have significant effects on R_2_* at t_0_ (Table 2).

**Figure 2:**
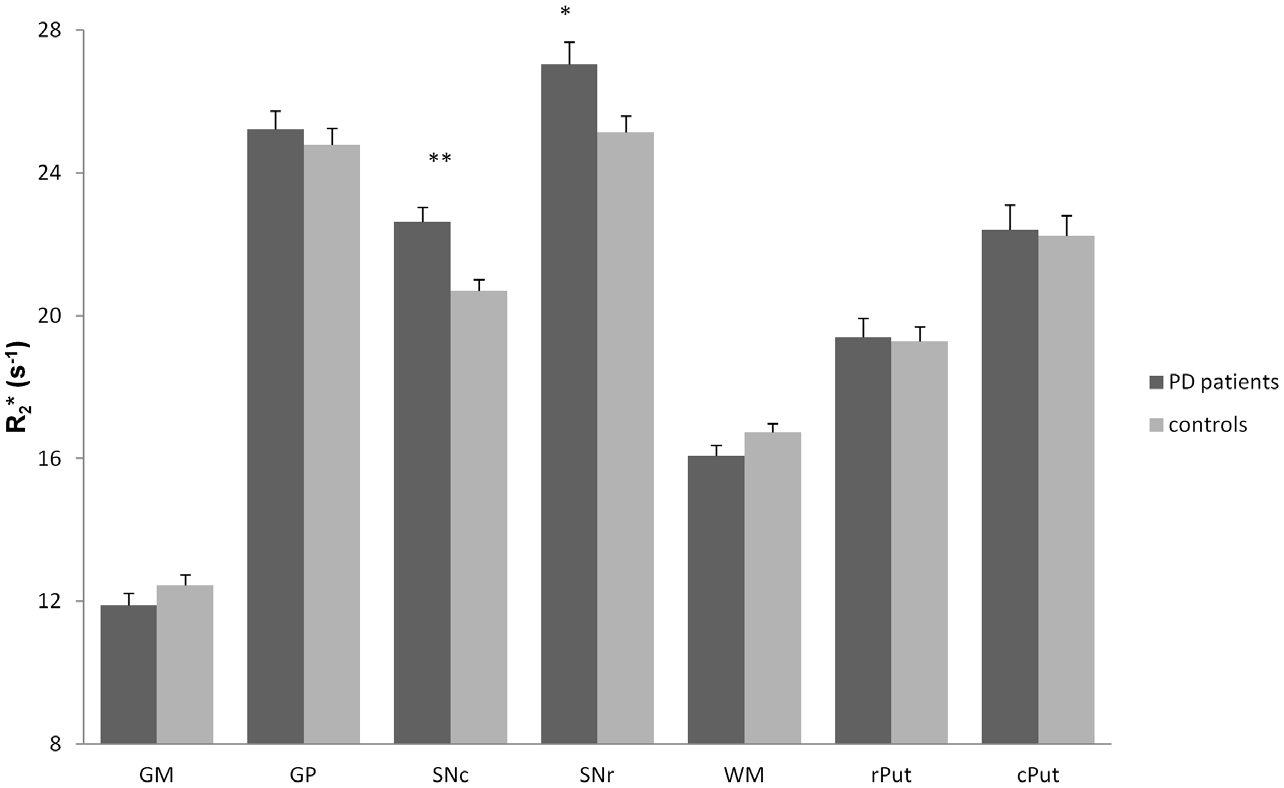
R_2_* (s^−1^) at t_0_ in each ROI (mean ± standard error) in PD patients and controls. GM = gray matter; GP = Globus Pallidus; SNc = Substantia nigra pars compacta; SNr = Substantia nigra pars reticulata; WM = white matter; rPut = rostral putamen; cPut = caudal putamen. **: p<0.001; *: p<0.05; analysis of variance (ANOVA).

Finally, the intergroup comparison at t_0_ with ANOVA showed a significant effect of PD on R_2_* in SNr (f = 6.55, p = 0.013) and SNc (f = 15.49, p<0.001), R_2_* being higher in the PD group. Also, R_2_* tended to be lower in the WM of PD patients (f = 3.08, p = 0.085) (Figure 2).

### Longitudinal Analysis of R_2_*

For controls, no significant evolution over 3 years was found. ΔR_2_* tended to be significant in WM with R_2_* decreasing by 0.82±0.44 s^−1^ (p = 0.099).

For PD patients, R_2_* increased by 2.32±0.63 s^−1^ (10.2%) in SNc (p = 0.001), 2.19±0.95 s^−1^ (8.1%) in SNr (p = 0.042) and 2.56±0.8 s^−1^ (11.4%) in the cPut (p = 0.011), while it decreased by 1.21±0.44 s^−1^ (7.5%) in WM (p = 0.042).

**Figure 3:**
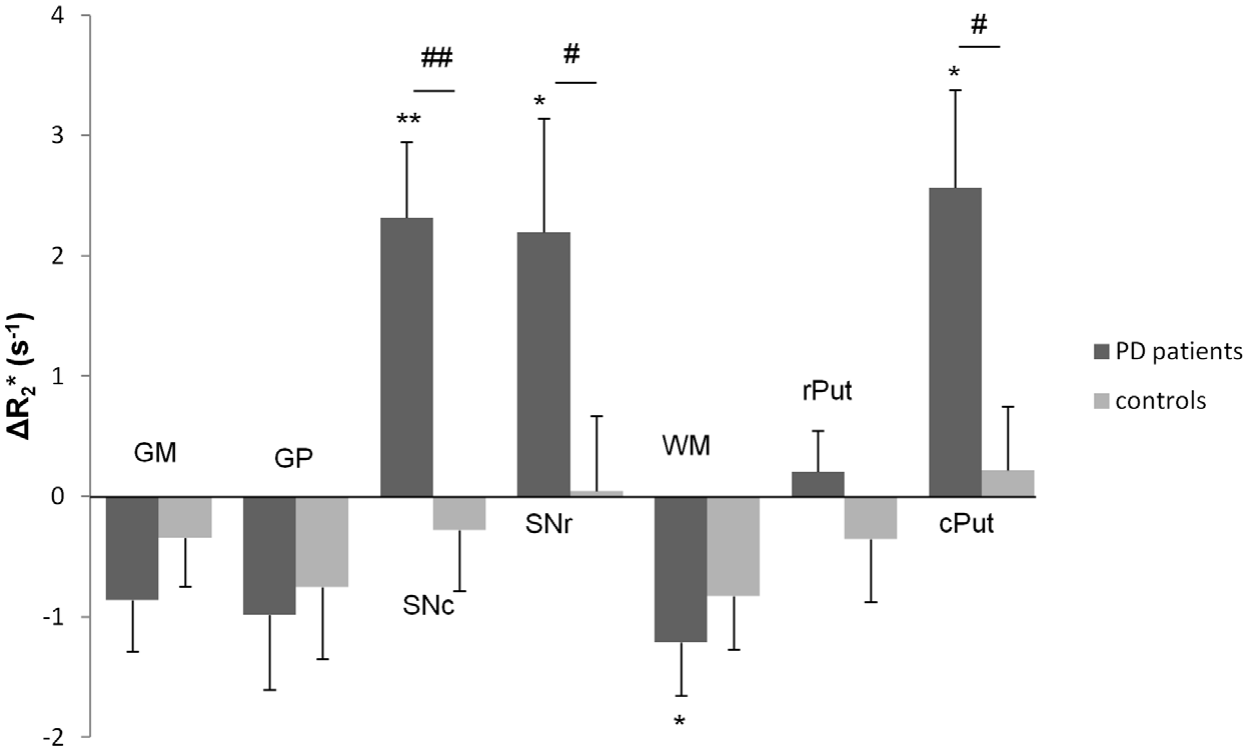
ΔR_2_* (s^−1^) in each ROI (mean ± standard error). A negative ΔR_2_*corresponds to a decrease of R_2_* after three years follow-up, whereas a positive ΔR_2_* corresponds to an increase of R_2_*. GM = gray matter; GP = Globus Pallidus; SNc = Substantia nigra pars compacta; SNr = Substantia nigra pars reticulata; WM = white matter; rPut = rostral putamen; cPut = caudal putamen. **: p<0.01; *: p<0.05 (significance of ΔR_2_* by group, signed rank test). ##: p<0.01; #: p<0.05 (effect of Parkinson’s disease on R_2_*, ANOVA).

Concerning the comparison between controls and PD patients, ΔR_2_* in PD changed significantly from that of controls in SNc (f = 12.1, p = 0.002), SNr (f = 5.05, p = 0.033) and in the cPut (f = 5.91, p = 0.022). It should be noted that there was no difference of ΔR_2_* between PD and controls in WM. The results of the longitudinal studies are grouped in Figure 3.

**Figure 4:**
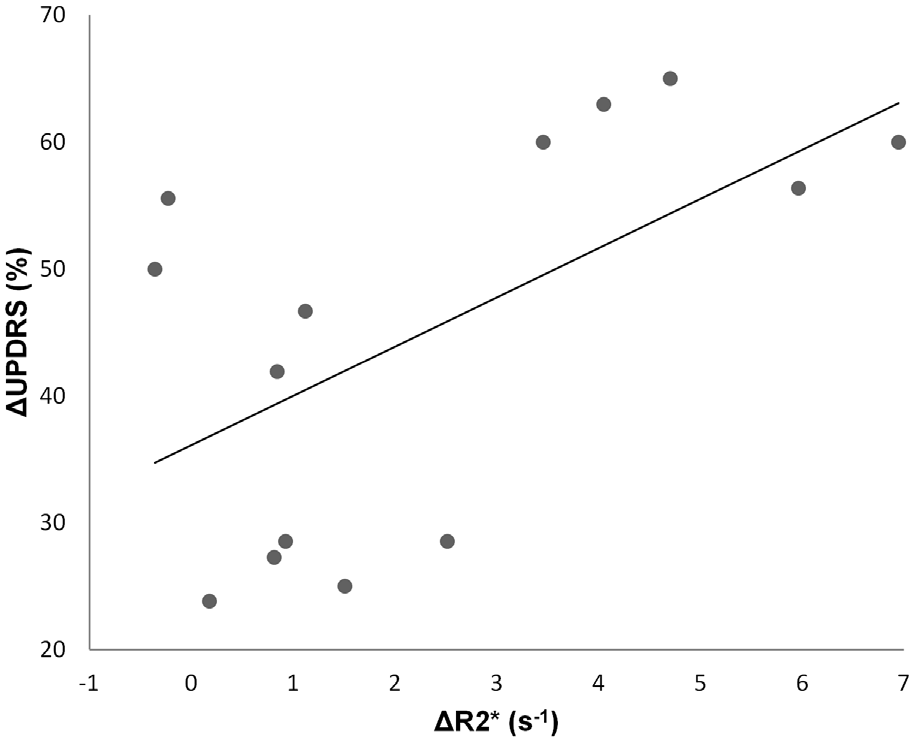
Correlation between ΔR2* and disease worsening. Scatter plot of the correlation between ΔR_2_* (s^−1^) and ΔUPDRS (%) in SNc showing a positive correlation (Spearman, R = 0.586, p = 0.028).

Finally, we found a positive correlation between ΔR_2_* and ΔUPDRS in SNc (R = 0.586, p = 0.028) and SNr (R = 0.608, p = 0.021) (Figure 4), but no correlation between ΔR_2_* and ΔHY nor between ΔR_2_* and ΔLED.

## Discussion

Using an adapted longitudinal analysis, we show for the first time: i- how quickly R_2_* increases due to the disease, since over 3 years it evolved by 10.2% in SNc, 8.1% in SNr and 11.2% in the cPut; ii- a positive correlation between ΔR_2_* and disease worsening assessed with motor part of UPDRS. We also confirm that R_2_* increased in the SN of PD patients compared to controls. Our results therefore suggest that R_2_*, previously considered as being a disease marker, is also sensitive to its evolution.

### Cross-sectional Analysis at t_0_

We confirm that R_2_* is significantly higher in the SN of PD patients and that no changes had occurred in the others parts of BG, in agreement with previous R_2_*-based cross-sectional studies [3], [7], [9]. Despite obvious anatomical differences between the two parts of the SN, few works have dealt with them separately. Only Martin *et al.* [5] analyzed these two structures, reporting an increase of R_2_* limited to the lateral part of the SNc, whereas we found a significant increase of R_2_* in both parts. This inconsistency could be explained by a different duration of the disease, 3 years on average in the study by Martin *et al*. instead of 6 years in our study. In addition, the lack of contrast between the two parts of the SN in the MR images must be emphasized, which introduces uncertainty in ROI positioning and thus may contribute to the differences between the two studies [24]. It should be noted, however, that we used the same method as Martin *et al.* to place the ROI in the 2 parts of the SN. We did not find any difference between less and most affected sides in the SN whereas a correlation between the lateralized UPDRS motor scores from the clinically most affected side and R_2_* values from the opposite SN was shown in early PD [5]. These differences could again be explained by the longer disease duration of our patients, leading to mainly symmetrical motor signs. Finally we showed no effect of disease duration and HY stage on R_2_* according to the literature [4], [5], [7], [9], [25]. There is considerable heterogeneity in the rate of progression of PD, which can be influenced by clinical, genetic and external factors, the relative contribution of these factors which remain incompletely understood [26], [27]. This interindividual variability in terms of rate of disease progression may explain why our cross-sectional study is insufficient to demonstrate a link between the variables which had been assessed (UPDRS, HY stage and disease evolution) and the R_2_*. This fact emphasizes the need to perform an individual and longitudinal approach to assess R_2_* as a biomarker of disease progression, the patient being his own control.

In the controls, we found an effect of age on R_2_* in the putamen (significant in the caudal part) and GM, with an increase in elderly subjects, in agreement with other cross-sectional studies [15]–[17], [28]. We also observed an increase of R_2_* with age in the putamen of PD patients, with results reaching significance only in the rostral part.

### Longitudinal Analysis

In PD patients, in which both aging and disease evolution are involved in the change of iron content estimated with R_2_*, longitudinal follow-up is the best way of taking these two factors into account. Thus it is likely that the disease itself is the main explanation for the rapid increase of R_2_* in the SN because no change of R_2_* due to aging was observed in this structure in the control group. In the caudal putamen, although cross-sectional analysis showed higher R_2_* in older controls, the longitudinal follow-up did not highlight any significant evolution over three years, demonstrating the predominant role of PD in increasing R_2_* in this structure.

The significant variation of R_2_* over a short period in structures involved in the physiopathology of PD [29], underlines the sensitivity of this parameter to disease evolution, since the HY stage and UPDRS used to evaluate the severity of the disease were significantly exacerbated over this same period of 3 years. Interestingly, we also found a positive correlation between ΔR_2_* and ΔUPDRS in SN, providing an additional argument in favor of ΔR_2_* as a biomarker of disease progression.

As the variation of R_2_* is essentially linked to modifications of iron content, our results suggest rapid accumulation of iron in the SN and the cPut during disease evolution. This rapid accumulation, particularly in the SN, suggests that iron plays a role in neuronal death mechanisms, in addition to its role as a vulnerability factor described by certain authors [13].

Several hypotheses [30] have been evoked to explain the increase of iron in the SN of PD patients, such as increased penetration of iron in dopaminergic neurons [31]–[33] and microglial activation [34], [35]. The migration of microglial cells in the SN, which represents a normal immune response to the degenerative process, could also participate in maintaining this process [35]. At the pathophysiological level, iron participates in the degenerative process as it contributes to the generation of reactive oxygen species inducing oxidative stress [36]. Works on animal models [37] show that the death of dopaminergic neurons precedes iron accumulation. Thus iron may not play a role in the initiation of the degenerative process but its increase as the disease worsening could indicate an important mechanism ensuring its persistence.

However, it is likely that the variations of R_2_* observed in this study do not exclusively reflect those of iron concentration. Indeed, the R_2_* relaxometry method worked well in brain tissues studied post mortem [29], but correlations observed between R_2_* and iron concentration was weaker in vivo [30], [31]. A large amount of R_2_ is non-iron dependent which makes R_2_* (R_2_* = R_2_+ R_2_’) sensitive to the neurodegenerative process that affects water content [38]. Furthermore, R_2_’ is very sensitive to brain oxygenation [39] and the arrangement at cellular scale of tissue constituents having different magnetic susceptibilities [18]. Thus we cannot discard the possibility that vascular or microstructural modifications of basal ganglia during the evolution of PD could participate in variations of R_2_* mainly due to iron accumulation. However, vascular involvement seems unlikely in our subjects, since we did not find any vascular lesion on MRI.

Lastly, we found a reduction of R_2_* in the WM of PD evaluated at 7.5% over 3 years, a point that has not yet been studied in PD. Siemonsen et *al.* [17], demonstrated a decrease of R_2_* correlated with normal aging, which could be linked with structural modifications of white matter described in elderly subjects, such as demyelination and axonal changes [17]. Interestingly, the decrease of R_2_* tended to be significant in our control group. Moreover, no difference was found between PD and controls in longitudinal follow-up. These two last points lead us to presume that age plays a predominant role in explaining the variations of R_2_* in the frontal WM observed in our PD patients.

In conclusion, we showed that a significant increase of R_2_* was observed longitudinally in substantia nigra and caudal putamen of PD patients, and correlated with clinical markers of disease worsening, emphasizing its interest as a biomarker of the disease’s evolution. The result of this longitudinal follow-up, collected from only 18 controls and 14 PD patients, asks to be confirmed on a larger cohort.

Our results suggest that such quantitative MRI relaxometry could be an interesting tool for an individual assessment of the progression of the neurodegeneration due to PD, and it would also be interesting for testing the efficiency of specific iron chelators and disease-modifying treatments. Finally, the sensitivity of relaxation rates to iron content is known to increase with the resonance frequency [20]. Consequently, the magnitude of R_2_* variations, over three years, which have been highlighted here at 1.5 T, would be presumably greater with higher magnetic field strength. This provides an opportunity to reduce the follow-up period separating two R_2_* measurements, a point which deserves further studies.
